# Hospital Officers: Their Training and Work

**Published:** 1912-03-23

**Authors:** 


### HOSPITAL OFFICERS: THEIR TRAINING AND WORK.

### The Ideal Hospital Chairman.

To begin with, the Chairman of a hospital board ought to understand his business and have a wonderfully reliable and retentive memory. It is not enough for him to be kind-hearted, intellectual, one quick in the "uptake" (as they say in Devonshire), one who can easily concentrate his thoughts and be readily able to assume an air of real authority; he must have many more than all these attributes. He must be a man whose largeness of heart will enable him to put himself in the place of others; a man of some imagination and of deep feeling. He must also be a man of resource, and one to whom the art of repartee comes naturally. Perhaps there are to a sensitive soul few human experiences more trying and more provoking than to take part in a business meeting under the chairmanship of one who is incompetent and dull-witted. A capable chairman makes a good board and sharp officials.

The chairman must have an "atmosphere," if he desires to be a success. He must be even-tempered, and it is all the better if he is good-humoured. If he should ever be so weak as to lose his temper at a meeting, he might as well leave the room with the business under consideration still unfinished. A chairman without amiability and strict impartialty is worse than no chairman at all. All good chairmen make it an invariable rule to stand by the secretary; they will not have him bullied. The chairman and the secretary (if the latter be worthy of the comradeship) should work together with as much accord as do the two men one sees with a long saw between them, cutting through a log of timber. The chairman should make it a rule to bar aimless questions about matters which are not on the agenda. He is the man at the wheel, and the secretary may be compared with the man in charge of the engines; the work of the one relies a good deal upon the work of the other. A good chairman will always *seem* to have time (and temper) enough to answer any silly question, yet at meetings where a dozen or so important affairs are down for discussion it is sheer cruelty to expect him to be everything, from a ready-reckoner to an obliging word-exchanger. If he has no leisure in the chair, no thoughts of value will be born to him.

The chief aim of a chairman should be to see that business is done thoroughly and with no waste of time. In order to do this, he must curb the exuberance of colleagues who do not keep to the point at issue. One good way of dealing with an immaterial question is to glance genially at the gentleman who has put it, and to continue the glance without saying a word. Patience on the part of a chairman is absolutely essential; he must suffer fools gladly; he must, when at a meeting, be polite even in the face of circumstances that in a railway carriage would richly deserve rude contradiction.

Debating power on the part of a chairman is desirable, but not nearly so necessary as the knack of weighing statements. A good chairman knows the idiosyncrasies of his colleagues, and he soon learns to attach the necessary importance to the different utterances of different men. He knows, for instance, that Mr. Smith really cannot be expected to agree with Mr. Brown, and he also knows that any amendment moved by Mr. Jones will find a stubborn seconder in Mr. Robinson.

Perhaps one of the most important gifts a chairman can possess is that which enables him to summarise a whole and long affair into a few pithy sentences. We all know the man who can talk for a quarter of an hour without managing to convey his meaning. A chairman should be lucid and brief; his aim should be straight and his words weighty. He should always appear to be master of the situation, and never afraid of any opinions of any man present at any meeting. A timid chairman will make for an unruly board. A chairman may be too cocksure and too extreme, and that fault is almost as serious as any other. It is the duty of a chairman to "keep the coach on the road" by sweet reasonableness and quiet persuasiveness. He should be ever ready to suggest a remedy, to seize quickly upon the pith of the discussion, and fasten upon the point that invites agreement.

A fair and simple preliminary test of a good hospital chairman is the manner in which he receives the matron of the place when she attends a meeting for the purpose of submitting a report, or for making a request of one sort or other. He will at once put her at her ease by his words of greeting; his very hand-shake will give her confidence; he will let her see that he thinks it very brave of her to come among a group of mere men who are sitting ready (and perhaps eager) to pounce upon any inept remark or any miscalculation. The chairman will take her under his wing, and she will not be afraid!

But there are no end to the traits of an ideal chairman. One might as well attempt to define a sunbeam as to put within a few lines of print all that one's mind conjures up on this interesting subject.

